# Early adjunctive vasopressin was not associated with a lower 7-day risk of renal replacement therapy in norepinephrine-treated patients with cirrhosis: a target-trial-informed study with external replication

**DOI:** 10.3389/fphar.2026.1941459

**Published:** 2026-09-23

**Authors:** Wei Zhang, Keyan Miao, Hui Chen, Gang Lu, Ziyang Zhou, Jianguo Tang

**Affiliations:** 1 Department of Trauma-Emergency and Critical Care Medicine, Shanghai Fifth People’s Hospital, Fudan University, Shanghai, China; 2 Department of Geriatrics, Shanghai Fifth People’s Hospital, Fudan University, Shanghai, China

**Keywords:** cirrhosis, critical care, norepinephrine, pharmacoepidemiology, real-world evidence, renal replacement therapy, target trial framework, vasopressin

## Abstract

**Background:**

Early adjunctive vasopressin can reduce catecholamine exposure in vasodilatory shock, but whether it prevents severe kidney failure in norepinephrine-treated patients with cirrhosis is uncertain.

**Methods:**

We conducted a target-trial-informed landmark study using MIMIC-IV as the primary cohort and eICU-CRD as an external replication cohort. Adults with a first intensive care unit admission, cirrhosis or advanced chronic liver disease, and norepinephrine initiation within 24 h of admission were eligible. Treatment was classified at a 6-h landmark as vasopressin initiated within 6 h of norepinephrine or no vasopressin during that window; patients in the latter group could receive vasopressin later. The primary outcome was new renal replacement therapy (RRT) between the landmark and day 7. Propensity-score overlap weighting was prespecified as the primary analysis.

**Results:**

The MIMIC-IV cohort included 1,086 patients (early vasopressin, n = 295; no early vasopressin, n = 791), and the eICU-CRD cohort included 356 patients (n = 91 and n = 265, respectively). Before weighting, vasopressin recipients had greater shock and liver-disease severity. Early vasopressin was not associated with lower odds of new RRT in MIMIC-IV (overlap-weighted odds ratio [OR] 1.37, 95% confidence interval [CI] 0.83–2.26; P = 0.214), eICU-CRD (OR 1.10, 95% CI 0.45–2.69; P = 0.833), or the pooled analysis (OR 1.30, 95% CI 0.84–2.01; P = 0.235). Mortality was higher among early-vasopressin recipients, but this secondary association remained highly vulnerable to confounding by indication. Weighted absolute-risk estimates, cumulative RRT incidence, subgroup analyses, and sensitivity analyses provided no supporting renal-benefit signal; creatinine trajectories were evaluated descriptively because follow-up measurements were susceptible to survivor selection.

**Conclusion:**

Among norepinephrine-treated patients with cirrhosis or advanced chronic liver disease who were alive and RRT-free 6 h after norepinephrine initiation, early adjunctive vasopressin was not associated with less RRT by day 7. The findings do not challenge vasopressin use for hemodynamic support when clinically indicated, but do not support early initiation solely as a renal-protective strategy.

## 1 Introduction

Cirrhosis and acute-on-chronic liver failure (ACLF) profoundly alter circulatory physiology. Splanchnic vasodilation, reduced effective arterial volume, systemic inflammation, and organ cross-talk make shock difficult to reverse and particularly hazardous to the kidney (Moreau et al., 2023; Arroyo et al., 2020). Acute kidney injury (AKI) in this setting is heterogeneous: hepatorenal syndrome–AKI, sepsis-associated tubular injury, hypovolemia, venous congestion, and drug-related injury may coexist (Nadim and Garcia-Tsao, 2023; Nadim et al., 2024). Infection is a common precipitant of acute decompensation and further magnifies circulatory and kidney dysfunction (Bajaj et al., 2021). Progression to renal replacement therapy (RRT) identifies a particularly vulnerable population and complicates decisions about transplant candidacy and goals of care, making prevention of severe kidney dysfunction a major clinical objective.

Vasopressin has a compelling pharmacological rationale in vasodilatory shock. Relative vasopressin deficiency contributes to loss of vascular tone, and exogenous replacement can increase arterial pressure while limiting catecholamine exposure (Landry et al., 1997). Small physiological trials in early septic shock also suggested improved urine output or creatinine clearance compared with norepinephrine (Lauzier et al., 2006). In cirrhosis, the efficacy of terlipressin plus albumin in selected patients with hepatorenal syndrome–AKI supports the possibility that vasopressin-pathway therapy could improve kidney perfusion (Wong et al., 2021). The analogy is incomplete, however. Patients with cirrhosis who require intensive care vasopressors frequently have infection, bleeding, mechanical ventilation, established organ failure, and structural as well as functional kidney injury. A rise in mean arterial pressure may therefore not translate into recovery of glomerular filtration or avoidance of RRT.

Current sepsis guidance recommends norepinephrine first and suggests adding vasopressin when arterial pressure remains inadequate (Evans et al., 2021). In VASST, adjunctive low-dose vasopressin did not improve mortality in the overall population (Russell et al., 2008). VANISH subsequently found no improvement in kidney failure–free days with early vasopressin, although fewer vasopressin-treated patients received RRT as a secondary outcome (Gordon et al., 2016). An individual-patient-data meta-analysis similarly found no mortality benefit, and its modest reduction in RRT was sensitive to analytic assumptions (Nagendran et al., 2019). Recent real-world studies have used increasingly rigorous causal-inference methods to examine vasopressor timing and escalation in septic shock, including target-trial and cross-fitted doubly robust approaches with external replication (White et al., 2025; Zhang et al., 2026). These studies do not resolve the cirrhosis-specific question because their populations, treatment contrasts, and principal outcomes differ.

Cirrhosis-specific evidence remains limited to a small cohort of catecholamine-resistant septic shock that found no mortality advantage for vasopressin over other second-line agents (Myc et al., 2017). Whether early adjunctive use is associated with less RRT in a broader population of norepinephrine-treated patients with cirrhosis is therefore uncertain. Routine observational comparisons are vulnerable to confounding by indication and time-related bias because vasopressin is preferentially given to patients with more refractory circulatory failure and treatment assignment evolves over time. Explicit alignment of eligibility, time zero, treatment classification, and outcome follow-up can reduce avoidable bias (Hernán and Robins, 2016). We therefore used a target-trial-informed 6-h landmark design to examine early adjunctive vasopressin and new RRT by day 7 in MIMIC-IV, with external replication in eICU-CRD. Relative estimates were complemented by weighted absolute risks, creatinine trajectories, RRT-free days, subgroup analyses, and sensitivity analyses.

## 2 Materials and methods

### 2.1 Study design and data sources

We conducted a retrospective, target-trial-informed landmark study using two deidentified critical-care databases. MIMIC-IV version 3.1 contains single-center hospital and intensive care unit data from Beth Israel Deaconess Medical Center (Johnson et al., 2023). eICU-CRD version 2.0 contains multicenter data from 208 United States hospitals and served as the external replication source (Pollard et al., 2018). Both resources are distributed through PhysioNet to credentialed researchers under data-use agreements (Goldberger et al., 2000). Reporting was guided by the STROBE and RECORD statements (von Elm et al., 2007; Benchimol et al., 2015).

The protocol aligned the clinical question with an explicit time zero, a 0–6 h treatment-classification window, a 6-h landmark, and outcome follow-up beginning only after treatment classification. The target-trial specification is summarized in Supplementary Table S1. Because this was a retrospective analysis of care already delivered and did not prospectively assign an intervention, clinical-trial registration was not applicable. The 6-h window was chosen to capture clinically early adjunctive vasopressor escalation while limiting the interval between T0 and the start of outcome follow-up.

Both databases contain deidentified data. The source-data approvals and waivers of informed consent are detailed in the Ethics statement; no additional patient contact or intervention occurred in this secondary analysis.

### 2.2 Study population, time zero, and treatment strategies

We identified adult patients with a first ICU admission, a prespecified diagnosis of cirrhosis or advanced chronic liver disease, and norepinephrine initiation within 24 h after ICU admission. Time zero (T0) was the first norepinephrine administration. Norepinephrine treatment operationalized circulatory failure for cohort entry; neither sepsis nor a specific shock etiology was required. The databases did not permit reliable, mutually exclusive adjudication of distributive, hypovolemic or hemorrhagic, cardiogenic, and mixed states. We therefore describe the study population as norepinephrine-treated patients with cirrhosis or advanced chronic liver disease rather than as a sepsis-defined shock cohort. We excluded patients receiving chronic dialysis or with end-stage kidney disease, patients who received vasopressin before T0, and patients who died, were discharged, received RRT, or lacked follow-up by the 6-h landmark. Cohort assembly is shown in Figure 1.

**FIGURE 1:**
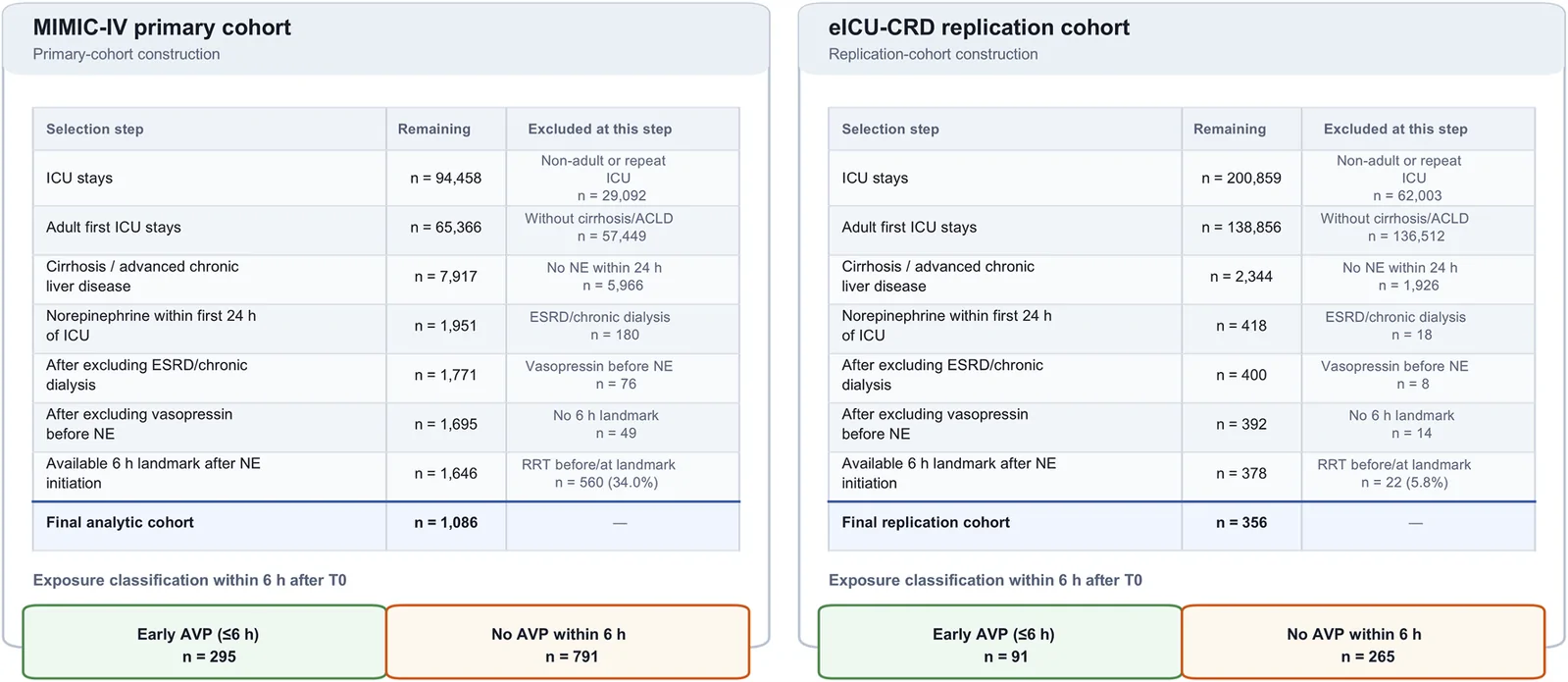
Cohort selection in MIMIC-IV and eICU-CRD. Before final cohort inclusion, RRT before or at the 6-h landmark excluded 560 of 1,646 MIMIC-IV patients (34.0%) and 22 of 378 eICU-CRD patients (5.8%); percentages use the number available at that selection step as the denominator. The final analytic cohorts comprised 1,086 patients in MIMIC-IV and 356 in eICU-CRD. ACLD, advanced chronic liver disease; AVP, vasopressin; ESRD, end-stage renal disease; ICU, intensive care unit; NE, norepinephrine; RRT, renal replacement therapy; T0, first norepinephrine administration.

Treatment was classified at the landmark from care delivered during the 0–6 h treatment-classification window. The early-vasopressin group comprised patients who initiated vasopressin at any time from T0 through 6 h; the comparator group comprised patients with no vasopressin during that window. Patients in the comparator group could receive vasopressin after the landmark, and subsequent treatment did not alter their exposure classification. The contrast is therefore early vasopressin versus no early vasopressin (early versus the rest), not early versus late or early versus never use. This design estimates the association with subsequent outcomes among patients who survived and remained RRT-free through 6 h; it does not estimate an effect among patients who died or required RRT during the classification window. The timeline is shown in Figure 2.

**FIGURE 2:**
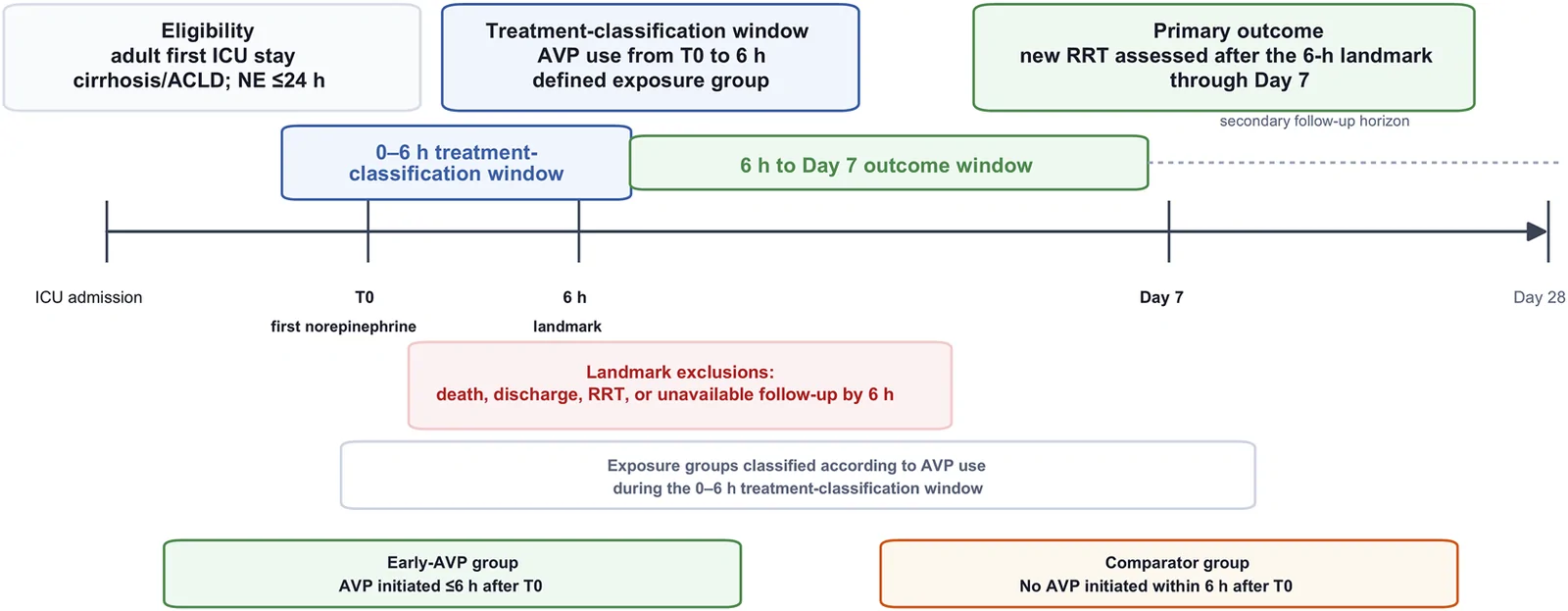
Target-trial-informed landmark design. Exposure was classified from vasopressin use during the 0–6 h treatment-classification window after T0. Patients with no vasopressin during that window could receive it after the landmark without changing classification. Primary-outcome follow-up extended from immediately after the landmark through day 7. ACLD, advanced chronic liver disease; AVP, vasopressin; ICU, intensive care unit; NE, norepinephrine; RRT, renal replacement therapy; T0, first norepinephrine administration.

A supplementary timing analysis described first vasopressin initiation after T0 in both databases. Initiation was categorized as 0–6 h, > 6–12 h, > 12–24 h, > 24–48 h, > 48 h–day 7, or no vasopressin recorded by day 7. This analysis quantified how often and when patients in the comparator group later received vasopressin and distinguished the prespecified no-early-vasopressin contrast from a never-vasopressin contrast.

### 2.3 Outcomes

The primary outcome was new RRT between the 6-h landmark and day 7. Database-specific procedure and treatment records were used to identify RRT, including continuous kidney replacement therapies and intermittent hemodialysis when recorded in either database. Modality-specific analyses were not performed because modality coding was not sufficiently consistent across sources. In the binary primary analysis, death before RRT was treated as no new RRT and was examined separately in the day-7 outcome-state analysis. Secondary outcomes were 28-day mortality, ICU mortality, hospital mortality, ICU and hospital length of stay, and RRT-free days through day 7. RRT-free days were the number of days alive without RRT between the landmark and day 7; patients who died before receiving RRT during this period were assigned 0 days. This measure therefore combines survival and renal outcome and should not be interpreted as kidney recovery alone. Because complete post-discharge mortality was unavailable in eICU-CRD, its 28-day outcome was defined as in-hospital death occurring within 28 days after norepinephrine initiation.

Weighted creatinine trajectories and the cumulative incidence of new RRT through day 7 were summarized descriptively as complementary analyses beyond the binary primary endpoint. A day-7 outcome-state analysis classified patients as alive without RRT, receiving new RRT, or dead before RRT.

### 2.4 Covariates and confounding control

Covariates were selected before outcome modeling because of their expected associations with vasopressin initiation, kidney injury, or both. They included age, sex, race or ethnicity, weight, liver-disease severity, a database-derived organ-dysfunction proxy, kidney function, creatinine, bilirubin, albumin, international normalized ratio, platelet count, sodium, lactate, mean arterial pressure, norepinephrine dose, mechanical ventilation, early albumin, corticosteroid and antibiotic exposure, cirrhosis-related complications, and major comorbidities. The organ-dysfunction proxy was a database-derived stratification variable and was not a clinically adjudicated ACLF diagnosis; it is renamed here to avoid implying equivalence to EASL-CLIF or other formal ACLF criteria. Baseline AKI stage was derived from available KDIGO-compatible data (Kellum and Lameire, 2013). Available-case denominators were used when the information needed to derive a variable was missing. Variables that could not be measured consistently in eICU-CRD were omitted from the replication model. Missing-data fractions for prespecified MIMIC-IV subgroup variables are reported with the subgroup results.

Because treatment was classified over the first 6 h, the propensity model used clinical information available by the landmark. Some hemodynamic summaries therefore covered the same window as exposure classification and were treated as landmark covariates rather than strictly pre-exposure measurements. This choice was made to compare patients with similar observed shock trajectories at the start of outcome follow-up; the resulting estimates were interpreted as associations rather than causal effects.

### 2.5 Statistical analysis

Continuous variables are reported as median (interquartile range), and categorical variables as number (percentage). Crude and multivariable logistic-regression models estimated odds ratios (ORs) with 95% confidence intervals (CIs) for new RRT. The prespecified primary analysis used propensity-score overlap weighting, assigning a weight of 1 minus the propensity score to early-vasopressin patients and the propensity score to comparators (Thomas et al., 2020). Overlap weighting emphasizes the population in whom either strategy was clinically plausible and limits the influence of extreme propensity scores. Covariate balance was evaluated with absolute standardized mean differences (SMDs), with 0.10 used as a practical threshold for residual imbalance (Austin, 2009). Observed cohort sizes are reported together with the full propensity-score and overlap-weight distributions because the weighted analysis targets an estimand-specific overlap population rather than a literal subset of retained patients.

We estimated weighted group-specific risks and risk differences for binary outcomes and weighted mean differences for continuous outcomes. The same prespecified analysis was repeated in eICU-CRD, and database-specific overlap-weighted ORs were combined with a fixed-effect inverse-variance model. Prespecified subgroup analyses examined baseline AKI stage, model for end-stage liver disease–sodium score, lactate, norepinephrine dose, mean arterial pressure, mechanical ventilation, and database-derived organ-dysfunction proxy; heterogeneity was assessed with treatment-by-subgroup interaction terms.

Sensitivity analyses used inverse probability of treatment weighting, doubly robust estimation, alternative covariate-adjusted models, and clinically defined cohort restrictions. These restrictions excluded patients with hepatorenal syndrome, gastrointestinal or variceal bleeding, or chronic kidney disease and separately restricted the cohort to database-derived organ-dysfunction proxy. All tests were two-sided, and P < 0.05 was considered statistically significant. Secondary, subgroup, and sensitivity analyses were interpreted as supportive rather than confirmatory because no multiplicity adjustment was applied. No *a priori* sample-size calculation was performed because all eligible records in each database were included.

To examine whether covariates measured during the 0–6 h treatment-classification window could partly reflect early treatment response, we performed a T0-only propensity-score sensitivity analysis. The model used information recorded at or before norepinephrine initiation, including demographics, comorbidities, cirrhosis-related complications, baseline laboratory values, baseline AKI stage, the database-derived organ-dysfunction proxy, mechanical ventilation, and norepinephrine dose at T0. Variables summarized during the 0–6 h window, including maximum norepinephrine dose, mean arterial pressure summaries, and post-T0 albumin or corticosteroid exposure, were excluded. Database-specific overlap-weighted estimates were supplemented by a pooled outcome model that included cohort as a covariate.

## 3 Results

### 3.1 Study population and baseline characteristics

Among 94,458 MIMIC-IV intensive care unit stays, 1,086 patients met all eligibility and landmark criteria; 295 received vasopressin within 6 h and 791 did not. At the final landmark-exclusion step, 560 of 1,646 patients (34.0%) were excluded because RRT had begun before or at the 6-h landmark. Among 200,859 eICU-CRD stays, 356 patients were included; 91 received early vasopressin and 265 did not. In eICU-CRD, 22 of 378 patients (5.8%) were excluded for RRT before or at the landmark (Figure 1). Baseline characteristics are provided in Supplementary Table S2 for MIMIC-IV and Supplementary Table S3 for eICU-CRD.

Before weighting, early-vasopressin recipients had higher lactate, greater norepinephrine exposure, lower minimum mean arterial pressure, more frequent mechanical ventilation, and more severe liver dysfunction. These differences are consistent with treatment escalation in patients with more refractory circulatory failure. Propensity-score and weight distributions showed limited but nonzero overlap (Supplementary Figure S1). Overlap weighting markedly improved measured covariate balance (Supplementary Figure S2), but the resulting inference pertains to a narrower overlap population and does not remove unmeasured differences. The two cohorts were not identical: the frequencies of chronic kidney disease and coded cirrhosis-related complications differed substantially, and spontaneous bacterial peritonitis was not recorded in eICU-CRD. The replication analysis therefore evaluates consistency across differently ascertained critical-care data rather than reproducing an identical case mix.

### 3.2 Primary renal outcome and external replication

In MIMIC-IV, new RRT occurred in 98 of 295 early-vasopressin patients (33.2%) and 176 of 791 comparators (22.3%). The crude OR was 1.74 (95% CI 1.29–2.33; P < 0.001). Adjustment moved the estimate toward the null, but the prespecified overlap-weighted analysis did not show lower RRT with early vasopressin (OR 1.37, 95% CI 0.83–2.26; P = 0.214).

In eICU-CRD, the corresponding unweighted incidences were 24.2% and 15.8%. The overlap-weighted OR was 1.10 (95% CI 0.45–2.69; P = 0.833). The pooled overlap-weighted OR was 1.30 (95% CI 0.84–2.01; P = 0.235). Database-specific and pooled estimates are displayed in Figure 3 and Table 1. None of the point estimates favored early vasopressin, although the CIs remained compatible with both modest benefit and harm.

**FIGURE 3:**
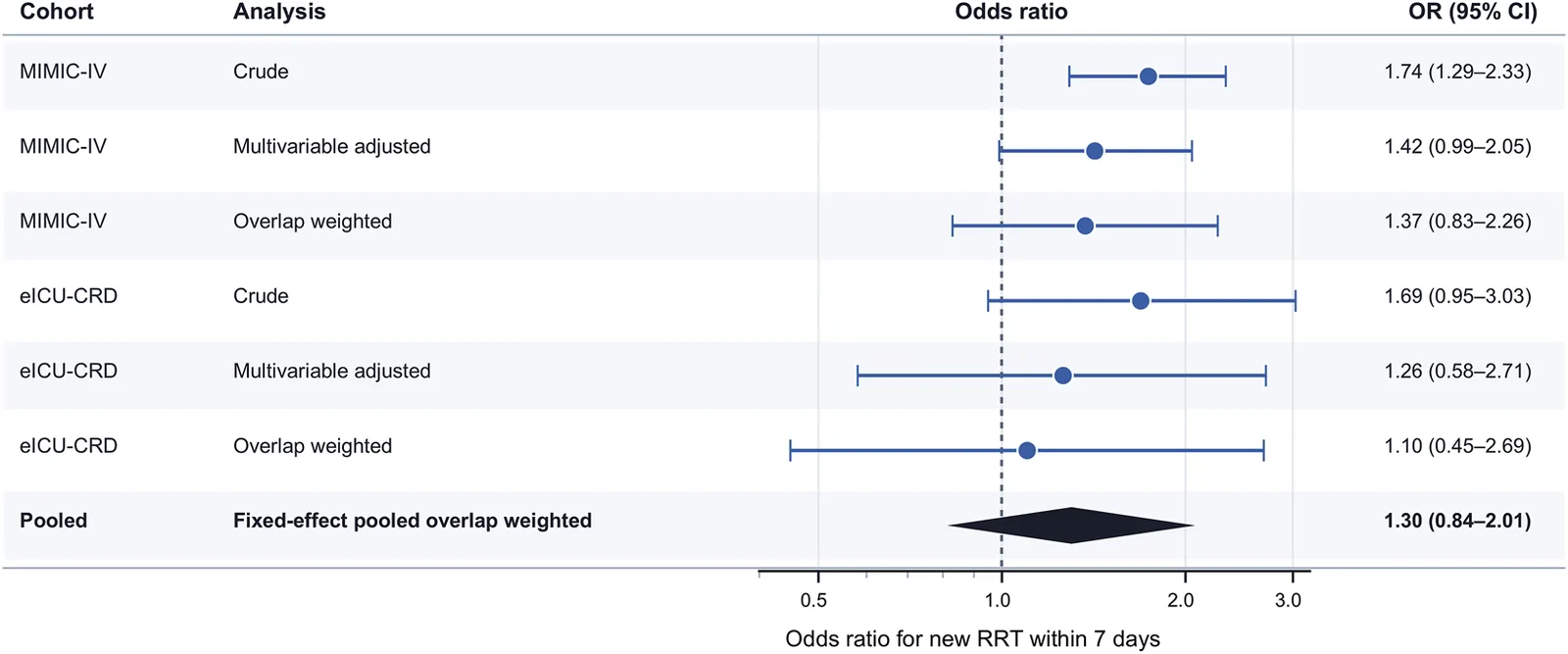
Early vasopressin and new renal replacement therapy. Odds ratios compare vasopressin initiated within 6 h with no vasopressin in that window; values greater than 1 indicate higher odds of new RRT with early vasopressin. The comparator group could receive vasopressin later. CI, confidence interval; eICU-CRD, eICU Collaborative Research Database; RRT, renal replacement therapy.

**TABLE 1:** Association between early vasopressin and new renal replacement therapy.

Dataset	Analysis	OR (95% CI)	P Value	N
MIMIC-IV	Crude	1.74 (1.29–2.33)	<0.001	1,086
MIMIC-IV	Conventional multivariable adjusted	1.42 (0.99–2.05)	0.059	1,086
MIMIC-IV	Overlap weighted	1.37 (0.83–2.26)	0.214	1,086
eICU-CRD	Crude	1.69 (0.95–3.03)	0.076	356
eICU-CRD	Conventional multivariable adjusted	1.26 (0.58–2.71)	0.559	356
eICU-CRD	Overlap weighted	1.10 (0.45–2.69)	0.833	356
Pooled	Fixed-effect pooled overlap weighted	1.30 (0.84–2.01)	0.235	1,442

Odds ratios compare early AVP with no early AVP. The overlap-weighted model was prespecified as the primary analysis. The conventional multivariable estimates in this table are distinct from the alternative adjusted sensitivity model shown in Supplementary Figure S6. AVP, Vasopressin; CI, Confidence Interval; eICU-CRD, eICU Collaborative Research Database; OR, Odds Ratio.

### 3.3 Absolute risk and kidney trajectories

In MIMIC-IV, the overlap-weighted risk of new RRT was 31.7% with early vasopressin and 25.3% without early vasopressin, a risk difference of 6.4 percentage points (95% CI −0.6 to 13.9). The corresponding eICU-CRD risks were 21.7% and 20.1%, a risk difference of 1.6 percentage points (95% CI −8.7 to 12.6). The pooled overlap-weighted risks were 28.9% and 23.8%, respectively, and the pooled risk difference was 5.1 percentage points (95% CI −1.2 to 10.8). These estimates are shown in Figure 4 and Supplementary Table S4.

**FIGURE 4:**
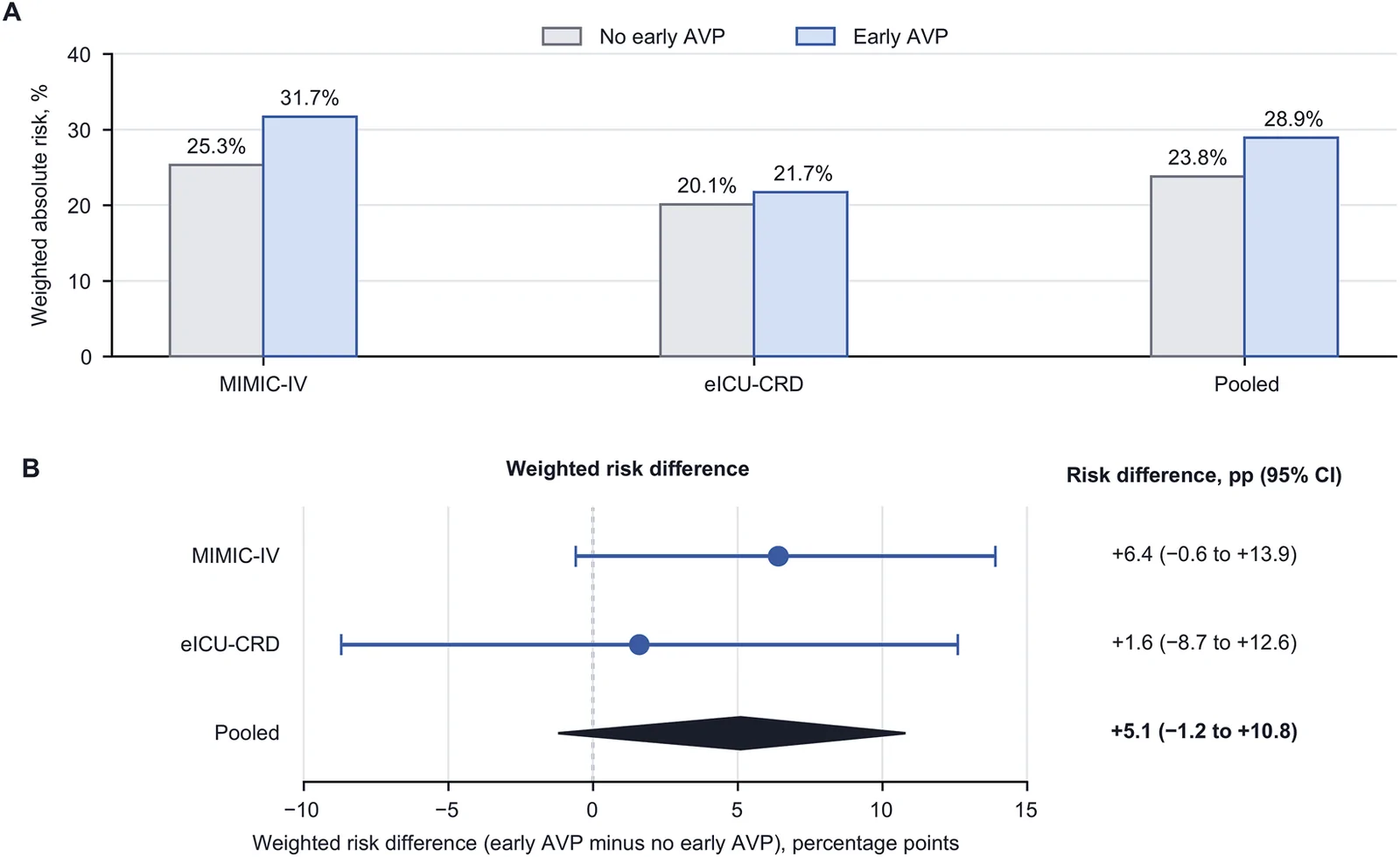
Overlap-weighted absolute risks and risk differences for new renal replacement therapy. Panel **(A)** shows database-specific and pooled risks. Panel **(B)** shows risk differences as early vasopressin minus no early vasopressin; positive values indicate higher risk with early vasopressin. Risk differences are expressed in percentage points (pp). AVP, vasopressin; CI, confidence interval; RRT, renal replacement therapy.

Weighted creatinine increased during the first 24–48 h in both MIMIC-IV groups and then declined (Figure 5). These trajectories are descriptive: the numbers contributing measurements at later time points differed between groups, mortality was also differential, and convergence of the curves may reflect survivor selection. They were therefore not used as evidence that a renal effect was absent. The weighted cumulative-incidence curves likewise did not show lower observed RRT after the landmark (Supplementary Figure S3).

**FIGURE 5:**
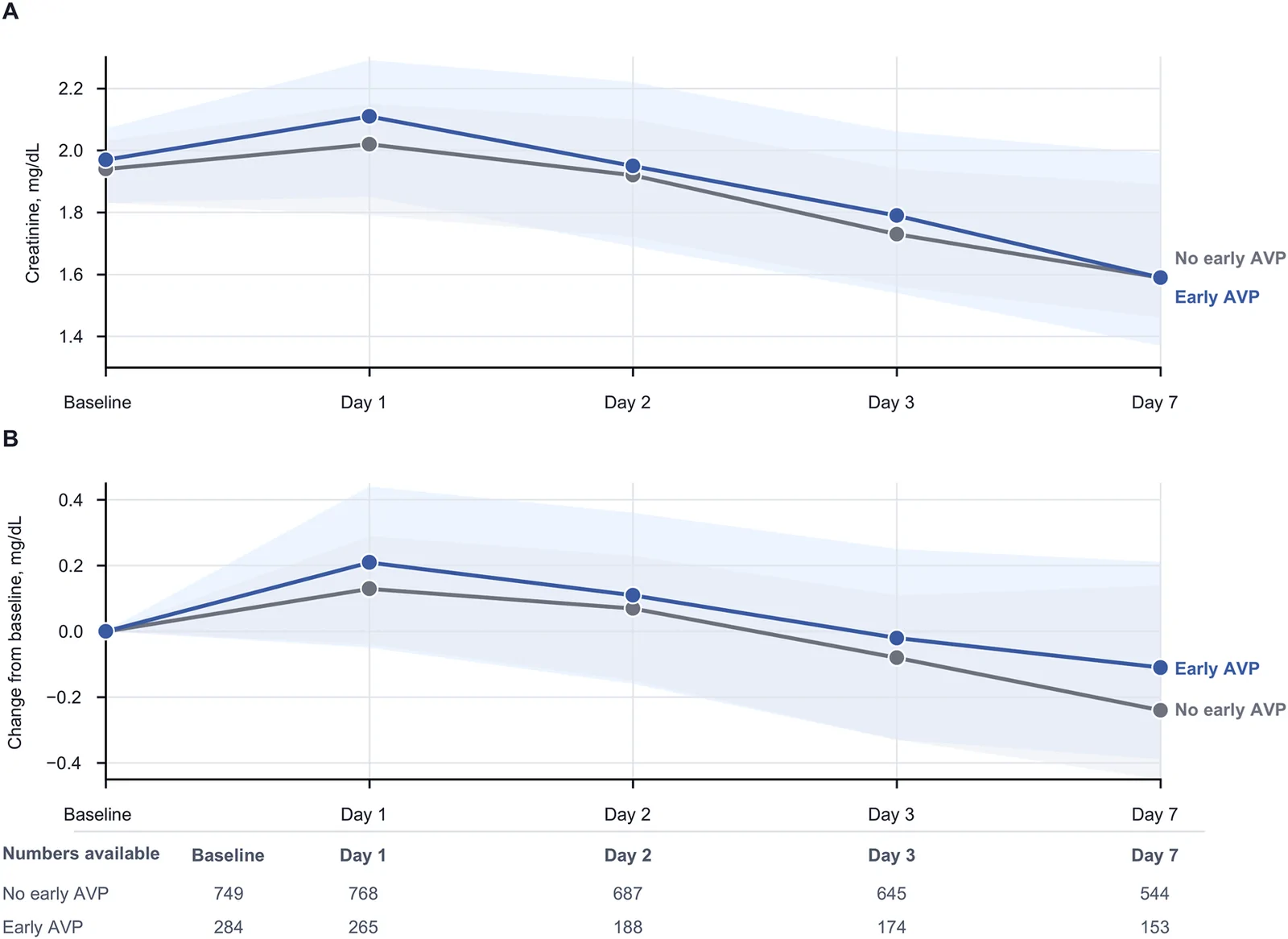
Descriptive overlap-weighted creatinine trajectories in MIMIC-IV. Panel **(A)** shows absolute creatinine concentrations, and panel **(B)** shows change from baseline. Lines show weighted means and shaded bands show bootstrap 95% confidence intervals. Later time points include only patients with available creatinine measurements; differential mortality and follow-up may therefore produce survivor selection, and convergence of the curves should not be interpreted as proof of no renal effect. AVP, Vasopressin; CI, Confidence Interval.

### 3.4 Secondary outcomes

Secondary outcomes are summarized in Supplementary Table S4. In MIMIC-IV, unweighted ICU mortality was 54.6% with early vasopressin and 31.4% without early vasopressin; 28-day mortality was 62.0% and 42.5%, respectively. The corresponding eICU-CRD proportions were 60.4% versus 35.8% for ICU mortality and 68.1% versus 45.7% for in-hospital death within 28 days. Mortality differences were attenuated but remained higher after overlap weighting. In MIMIC-IV, early vasopressin was also associated with fewer overlap-weighted RRT-free days through day 7 (3.25 vs. 3.86 days; weighted difference -0.61 days, 95% CI -1.12 to -0.11). These secondary associations were not adjusted for multiple comparisons and were considered hypothesis-generating because residual confounding by shock severity remained plausible.

Death competed strongly with RRT. In the binary primary outcome, death before RRT was counted as no new RRT; the higher mortality among early-vasopressin recipients therefore reduced their opportunity to receive RRT and would tend to attenuate, rather than create, an excess RRT association. The day-7 outcome-state analysis did not show a larger proportion alive without RRT among early-vasopressin recipients in either database (Supplementary Figure S4). RRT-free days should be read as a combined survival-and-renal measure because deaths were assigned 0 days. These descriptive analyses support the absence of a renal-benefit signal but do not eliminate competing-risk or treatment-selection bias.

### 3.5 Subgroup and sensitivity analyses

No prespecified subgroup had statistically significant evidence of a different association between early vasopressin and new RRT. Estimates were broadly similar across strata of AKI stage, MELD-Na, lactate, norepinephrine dose, mean arterial pressure, mechanical ventilation, and database-derived organ-dysfunction proxy; all interaction P values exceeded 0.05 (Supplementary Figure S5). These analyses had limited precision and should not be interpreted as proof of homogeneity. Baseline AKI stage and MELD-Na were missing for 39.1% and 38.4% of MIMIC-IV patients, respectively; these subgroup estimates require particular caution (Supplementary Table S5).

Sensitivity analyses produced the same qualitative conclusion. IPTW, doubly robust estimation, conventional adjustment, clinically restricted cohorts, eICU-CRD replication, and pooled estimation did not identify a lower RRT risk with early vasopressin (Supplementary Figure S6).

The supplementary timing analysis showed that later vasopressin initiation was common among patients classified in the no-early-vasopressin comparator group. In MIMIC-IV, 206 of 791 comparators (26.0%) initiated vasopressin after 6 h, with a median initiation time of 15.0 h (IQR 9.7–33.7); 585 (74.0%) had no vasopressin recorded by day 7. In eICU-CRD, 81 of 265 comparators (30.6%) initiated vasopressin after 6 h, with a median initiation time of 16.2 h (IQR 10.0–33.1); 184 (69.4%) had no vasopressin recorded by day 7 (Supplementary Table S6; Supplementary Figure S7). Thus, the comparator represented no vasopressin during the prespecified first 6 h, with later use permitted, rather than a never-vasopressin group.

The T0-only propensity-score sensitivity analysis yielded an overlap-weighted OR for new RRT within 7 days of 1.43 (95% CI 0.87–2.35; P = 0.157) in MIMIC-IV and 1.37 (95% CI 0.55–3.42; P = 0.503) in eICU-CRD. The pooled cohort-adjusted OR was 1.42 (95% CI 0.92–2.19; P = 0.118). These estimates were directionally consistent with the primary analysis and did not indicate a lower RRT risk with early vasopressin (Supplementary Table S7; Supplementary Figure S8).

## 4 Discussion

In this target-trial-informed landmark study, early adjunctive vasopressin was not associated with a lower risk of new RRT by day 7 among norepinephrine-treated patients with cirrhosis or advanced chronic liver disease who were alive and RRT-free 6 h after norepinephrine initiation. The crude excess of RRT among vasopressin recipients was attenuated substantially after overlap weighting, consistent with treatment selection toward patients with more severe circulatory failure. The eICU-CRD replication estimate pointed in the same direction, and neither weighted absolute risks nor the pooled analysis favored early vasopressin. No supporting renal-benefit signal emerged from the cumulative-incidence, Day-7 outcome-state, subgroup, or sensitivity analyses. Creatinine trajectories were treated as descriptive because later measurements were susceptible to survivor selection. The pooled confidence interval nevertheless included a modest benefit as well as clinically relevant harm, so the findings should not be interpreted as proof of equivalence or absence of any treatment effect.

The comparison with randomized evidence requires more nuance than the neutral primary outcomes alone suggest. VASST found no overall mortality advantage with adjunctive vasopressin (Russell et al., 2008), but a subsequent renal analysis suggested less progression to severe kidney failure and less RRT among patients who entered in the RIFLE Risk category (Gordon et al., 2010). VANISH did not improve kidney failure–free days, yet RRT was used in 25.4% of vasopressin-treated patients and 35.3% of norepinephrine-treated patients; RRT was a secondary outcome and its initiation was not protocolized (Gordon et al., 2016). An individual-patient-data meta-analysis subsequently estimated a small reduction in RRT, but the result was not robust to sensitivity analyses and there was no mortality benefit or convincing interaction by baseline AKI, lactate, norepinephrine dose, or treatment timing (Nagendran et al., 2019). Our results do not invalidate those signals; rather, they show that a possible renal effect in general septic shock cannot be assumed to extend to patients with cirrhosis, mixed shock states, and frequent pre-existing kidney dysfunction. The treatment contrasts also differ: VANISH evaluated early vasopressin as initial vasopressor therapy, whereas our study compared adjunctive vasopressin within 6 h with no vasopressin during that window, allowing later use in the comparator group. Cross-study effect-scale comparisons require caution; nevertheless, the lower bound of the pooled 95% CI in our study (OR 0.84) excludes an odds reduction of the magnitude implied by the RRT proportions reported in VANISH (25.4% vs. 35.3%; approximate OR 0.62). A smaller renal benefit remains compatible with our interval.

Recent observational studies have generally favored earlier rather than later vasopressin, but their estimands differ from ours. Higher norepinephrine dose and lactate at vasopressin initiation were associated with greater mortality in a two-database cohort (Sacha et al., 2022), and a MIMIC analysis linked initiation at lower norepinephrine doses to lower mortality (Xu et al., 2023). A multicenter Australian cohort reported lower hospital mortality with initiation within 6 h (White et al., 2024), and a later target-trial emulation from the same health system estimated a small 30-day intensive care unit mortality benefit (White et al., 2025). A recent severity-stratified, cross-fitted doubly robust analysis with external replication further illustrates the growing use of real-world data to study vasopressor escalation (Zhang et al., 2026). These studies focused on septic shock and principally on mortality or escalation strategies; several compared early with late administration only among patients who ultimately received vasopressin. Such analyses may identify a preferable time to treat among treated patients without establishing that an early strategy prevents RRT relative to no early vasopressin. Cirrhosis also introduces portal hypertension, altered effective circulating volume, bleeding, cardiomyopathy, infection-related inflammation, and a high burden of chronic or recurrent kidney dysfunction. The only prior cirrhosis-specific cohort included 45 patients with catecholamine-resistant septic shock and found no mortality advantage for vasopressin over other second-line agents (Myc et al., 2017).

Two recent systematic reviews further illustrate the uncertainty. One reported lower short-term mortality and less RRT with vasopressin initiated within 6 h, but the contributing studies were judged to be at high risk of bias (Bhattacharjee et al., 2025). A subsequent review found no reduction in mortality or RRT and likewise identified substantial risk of bias (Mamede et al., 2026). The discordance underscores why the treatment comparator, target population, timing definition, and control of confounding by indication are central to pharmacoepidemiological interpretation of apparent benefits from earlier use.

Several mechanisms could explain why improved vascular tone did not translate into less RRT. Kidney dysfunction in decompensated cirrhosis spans functional renal vasoconstriction, sepsis-associated tubular injury, hypovolemia, nephrotoxic exposure, venous congestion, and intra-abdominal hypertension (Nadim and Garcia-Tsao, 2023; Nadim et al., 2024). Critical-care guidance and cohort data emphasize that circulatory failure, infection, and nonrenal organ failures often evolve together rather than as separable processes (Nanchal et al., 2020; Bernal et al., 2021; Piano et al., 2019). Vasopressin may restore arterial pressure and reduce norepinephrine requirements without correcting inflammation, microcirculatory dysfunction, renal venous pressure, or established structural injury. The response to terlipressin plus albumin in carefully selected hepatorenal syndrome–AKI therefore cannot be extrapolated directly to heterogeneous intensive care shock (Biggins et al., 2021). Timing may also be phenotype-dependent: a patient with predominantly functional renal hypoperfusion might benefit before severe AKI is established, whereas the same intervention may offer little renal recovery once tubular injury or multiorgan failure dominates. We found no convincing interaction by baseline AKI, lactate, norepinephrine dose, mean arterial pressure, mechanical ventilation, or database-derived organ-dysfunction proxy, but the subgroup estimates were imprecise and do not exclude benefit in a narrowly defined phenotype.

The mortality signal warrants separate attention. In MIMIC-IV, ICU mortality was 54.6% versus 31.4% and 28-day mortality was 62.0% versus 42.5% for early versus no early vasopressin; the same direction, with larger weighted differences, was seen in eICU-CRD. We do not interpret these observational associations as evidence that vasopressin caused death. Early-vasopressin recipients had higher lactate, more norepinephrine exposure, lower arterial pressure, greater liver dysfunction, and more mechanical ventilation before weighting. Electronic health records also omit reasons for escalation, including capillary refill, echocardiographic findings, dynamic fluid responsiveness, evolving bleeding, and concern about impending collapse. Similar mortality patterns in two databases are compatible with the same treatment-selection process operating in both settings, and weighting cannot remove time-varying or unrecorded severity. That residual severity could also bias the RRT association upward and conceal a modest renal benefit. In the opposite direction, death before RRT shortened the opportunity to initiate RRT and could bias the observed RRT association downward. Because the magnitude of these opposing biases is unknown, the mortality difference cannot validate the renal estimate, and the RRT estimate should not be read as evidence of nephrotoxicity. The study does not demonstrate a renal benefit or establish causal harm.

RRT is clinically important but is not a purely biological marker of kidney failure. Initiation depends on potassium and acid-base disturbances, fluid accumulation, urine output, hemodynamic tolerance, transplant candidacy, goals of care, and local practice. In a cohort with 40%–60% mortality, the endpoint also partly reflects whether escalation to RRT was considered appropriate and consistent with the treatment plan. This dependence may reduce RRT use in some of the sickest patients and makes a pharmacological renal benefit harder to isolate. Failure to recover from AKI is common in critically ill patients with cirrhosis and is strongly associated with mortality (Worden et al., 2023). Trials of accelerated versus standard RRT initiation have shown that treatment exposure can vary markedly by initiation strategy without improving survival (Bagshaw et al., 2020). Among patients with cirrhosis who start RRT, transplant listing status strongly influences prognosis (Allegretti et al., 2018), and population-based data suggest that only about one-quarter recover sufficient kidney function to discontinue dialysis, usually within the first 3 months (Wang et al., 2022). Our day-7 endpoint reduces contamination by late complications but may miss delayed renal recovery or RRT after the first week. Death before RRT was treated as no event in the primary binary analysis; the day-7 state analysis separated death before RRT, whereas RRT-free days assigned 0 days to death and therefore conflated survival with renal outcome. These complementary analyses clarify, but cannot eliminate, competing-event and treatment-selection bias.

Clinically, these findings should not discourage vasopressin when an adjunctive vasopressor is needed to achieve hemodynamic targets or limit catecholamine exposure (Evans et al., 2021). They argue against initiating it early primarily as a renal-protective intervention in an unselected cirrhosis population. Kidney protection remains a bundle rather than a single-drug strategy: prompt treatment of infection or bleeding, judicious fluid and albumin administration, avoidance of nephrotoxins, assessment of venous congestion, repeated evaluation of AKI phenotype, and timely discussion of transplant suitability and goals of care (Nadim et al., 2024; Nanchal et al., 2020; Biggins et al., 2021). A future trial would be most informative if it enrolled a clinically coherent phenotype, standardized the trigger and dose for vasopressin, measured hemodynamic and congestion variables before treatment assignment, and adjudicated indications for RRT.

The timing and T0-only analyses refine the interpretation of the study. The timing distribution shows that the emulated contrast was not early versus never vasopressin, but early adjunctive vasopressin versus withholding vasopressin during the initial 6-h norepinephrine-treated window while allowing later initiation. A neutral result under this estimand argues against routine early initiation as a kidney-protective strategy but does not preclude later escalation for hemodynamic indications. The T0-only propensity model addresses the concern that 0–6 h covariates might partly lie downstream of early treatment. Its similar point estimates reduce the likelihood that the primary result was driven solely by adjustment for landmark-period response variables, although neither analysis removes residual confounding.

This study has several strengths. Eligibility, time zero, treatment classification, landmark exclusions, and outcome follow-up were aligned before modeling; the primary analysis was repeated in an independent multicenter database; and overlap weighting focused inference on patients with observed clinical equipoise. Relative estimates were accompanied by absolute risks and complementary kidney outcomes rather than a single adjusted regression result. Important limitations remain. Sepsis was not required, shock etiology was not adjudicated, and direct measures of intravascular volume, fluid responsiveness, active blood loss, and hemodynamic phenotype were unavailable. The cohort may therefore combine distributive, hypovolemic or hemorrhagic, cardiogenic, and mixed circulatory failure. Residual confounding is unavoidable, and several hemodynamic covariates were summarized during the same 0–6 h window used to classify treatment, so they may reflect early response as well as pretreatment severity. The T0-only analysis addresses this concern only partially; the estimates remain landmark associations rather than causal effects. Overlap was limited, the overlap estimand represents a narrower population than the full cohort, and incomplete covariate capture could not be assumed to be missing at random across two heterogeneous databases. We did not add a multiple-imputation analysis because a common imputation model would impose unverifiable cross-database assumptions and would not address unmeasured confounding. RRT indications, fluid balance, echocardiography, vasopressin dose, transplant status, and treatment limitations were incompletely measured. The eICU-CRD cohort had less granular medication and laboratory data, a smaller sample, different frequencies of chronic kidney disease and cirrhosis-related diagnoses, and no recorded spontaneous bacterial peritonitis. Concordance across these differently ascertained cohorts is a robustness check rather than evidence that they represent the same population. Conditioning on survival without RRT to 6 h also selected a lower-risk landmark population: 560 of 1,646 otherwise eligible MIMIC-IV patients (34.0%) and 22 of 378 eICU-CRD patients (5.8%) were excluded because RRT had already begun by the landmark. The findings therefore do not apply to the earliest or most rapidly progressive kidney events. Finally, fixed exposure classification does not represent a dynamic bedside strategy, and observational data cannot determine whether a different trigger, dose, or phenotype-specific approach would improve renal outcomes.

## 5 Conclusion

Among norepinephrine-treated patients with cirrhosis or advanced chronic liver disease who were alive and RRT-free at a 6-h landmark, early adjunctive vasopressin was not associated with a lower risk of new RRT by day 7. The pooled interval excluded a renal effect as large as that implied by the RRT proportions reported in VANISH, although a smaller benefit cannot be ruled out. The direction of the result was consistent in the eICU-CRD replication cohort and in the timing and T0-only sensitivity analyses. Vasopressin may remain appropriate as a hemodynamic adjunct when clinically indicated, but these data do not support early initiation solely to prevent RRT and do not establish a causal mortality effect.

## Data Availability

Publicly available datasets were analyzed in this study. This data can be found here: PhysioNet (credentialed access): MIMIC-IV version 3.1, https://physionet.org/content/mimiciv/3.1/ (DOI: 10.13026/kpb9-mt58); eICU Collaborative Research Database version 2.0, (DOI: 10.13026/C2WM1R). Access requires PhysioNet credentialing, completion of the required human-subjects training, and acceptance of the applicable data-use agreements.
