# Supplementary material for: Early adjunctive vasopressin was not associated with a lower 7-day risk of renal replacement therapy in norepinephrine-treated patients with cirrhosis: a target-trial-informed study with external replication
Source: Front Pharmacol. 2026 Sep 23;17:1941459. doi: 10.3389/fphar.2026.1941459 (PMC13642645; doi:10.3389/fphar.2026.1941459)
Supplement: Supplementary file 1 [file Supplementaryfile1.docx]

Supplementary Material

# 1 Supplementary methods

**Table S1.** Target-trial specification

| **Component** | **Operational specification** |
| --- | --- |
| Clinical question | Among norepinephrine-treated adults with cirrhosis or advanced chronic liver disease, was vasopressin initiation within 6 h associated with a lower risk of renal replacement therapy (RRT) after the 6-h landmark? |
| Data sources | MIMIC-IV version 3.1 for the primary cohort; eICU-CRD version 2.0 for external replication. |
| Eligibility | Adult first ICU admission; cirrhosis or advanced chronic liver disease; first norepinephrine administration within 24 h after ICU admission. Sepsis and a specific shock etiology were not required. |
| Time zero | First norepinephrine administration (T0). |
| Treatment-classification window | T0 through 6 h after T0. |
| Early-vasopressin strategy | Vasopressin initiated during the 0–6 h window. |
| Comparator strategy | No vasopressin during the 0–6 h window. Vasopressin could be initiated after the landmark without changing exposure classification. |
| Landmark exclusions | Chronic dialysis or end-stage kidney disease; vasopressin before T0; death, discharge, RRT, or unavailable follow-up by the 6-h landmark. |
| Exposure classification | Classified once at the 6-h landmark according to care received during the treatment-classification window. Subsequent treatment did not change classification. |
| Start of outcome follow-up | Immediately after the 6-h landmark. |
| End of primary follow-up | Day 7 after T0. |
| Primary outcome | New RRT after the 6-h landmark through Day 7. |
| Target population for inference | Patients who were alive, still under observation, and RRT-free at the 6-h landmark. |
| Primary contrast | Association of early vasopressin versus no early vasopressin (early versus the rest, not early versus late or never use) with new RRT. |
| Primary analysis | Propensity-score overlap-weighted logistic regression; primary analysis in MIMIC-IV followed by external replication in eICU-CRD. |
| Additional analyses | Weighted absolute risks and risk differences, descriptive creatinine trajectories, cumulative RRT incidence, secondary outcomes, prespecified subgroups, sensitivity analyses, fixed-effect pooling of the primary estimates, timing of first vasopressin initiation, and a T0-only propensity-score sensitivity analysis. |

## 1.1 Landmark covariates and interpretation

Treatment was classified from care delivered between norepinephrine initiation and the 6-h landmark. The propensity models used clinical information available by that landmark. Because some hemodynamic summaries covered the same window as treatment classification, they may capture early treatment response as well as illness severity. All estimates were therefore interpreted as landmark associations rather than causal effects.

## 1.2 Study population and shock phenotype

Norepinephrine initiation was used to identify clinically important circulatory failure. Sepsis was not required, and the databases did not permit reliable adjudication of mutually exclusive shock etiologies. The analytic population may therefore include distributive, hypovolemic or hemorrhagic, cardiogenic, and mixed states. Direct measures of intravascular volume, dynamic fluid responsiveness, active bleeding, and bedside echocardiography were not consistently available.

## 1.3 Outcome ascertainment and competing events

RRT was identified from database-specific procedure and treatment records and included continuous kidney replacement therapy and intermittent hemodialysis when recorded. Modality-specific analyses were not performed because coding was not sufficiently consistent across databases. In the primary binary outcome, death before RRT was treated as no new RRT. Day-7 outcome states separately classified patients as alive without RRT, receiving new RRT, or dead before RRT. Patients who died before RRT were assigned 0 RRT-free days; RRT-free days therefore combine survival and renal outcome.

## 1.4 Database-derived organ-dysfunction proxy

The database-derived organ-dysfunction proxy was used only for stratification and sensitivity restriction. It was not a prospectively adjudicated ACLF diagnosis and did not reproduce EASL-CLIF or another formal ACLF definition. Its high prevalence in this vasopressor-selected cohort also limited clinical specificity. The term database-derived organ-dysfunction proxy is used consistently in the revised text, tables, and figure panels.

## 1.5 Weighting and additional analyses

For overlap weighting, patients who received early vasopressin were assigned a weight of 1 minus the estimated propensity score, and comparators were assigned the propensity score. Covariate balance was assessed using absolute standardized mean differences, with 0.10 used as a practical threshold. The propensity-score and weight distributions demonstrate limited but nonzero overlap; no observations were trimmed. Observed cohort sizes are reported with the full distributions rather than as a pseudo-count because overlap weights define an estimand-specific target population rather than a literal retained subset. The analysis was performed first in MIMIC-IV and then repeated in eICU-CRD; database-specific overlap-weighted odds ratios were combined using fixed-effect inverse-variance pooling. Secondary, subgroup, and sensitivity analyses were considered supportive because no multiplicity adjustment was applied.

## 1.6 Database harmonization

Variables that could not be measured consistently in eICU-CRD were omitted from the replication model. Descriptive percentages used available-case denominators when source information needed to derive a variable was missing. Missingness was driven partly by database structure and partly by clinically selective measurement, so a common missing-at-random assumption across the two sources was not considered defensible. A new multiple-imputation analysis was therefore not added. Availability and missing fractions for prespecified MIMIC-IV subgroup variables are reported with the subgroup analyses. Database-specific baseline variables available in eICU-CRD are reported with the baseline tables.

# 2 Supplementary results

**Figure S1.** Propensity-score and overlap-weight diagnostics. Distributions in MIMIC-IV and eICU-CRD showed overlap without domination by extreme weights. AVP, vasopressin; eICU-CRD, eICU Collaborative Research Database.

## 2.1 Timing and T0-only sensitivity analyses

Among patients in the no-early-vasopressin comparator group, 206 of 791 MIMIC-IV patients (26.0%) and 81 of 265 eICU-CRD patients (30.6%) initiated vasopressin after 6 h. The median initiation times among these later initiators were 15.0 h (IQR 9.7–33.7) and 16.2 h (IQR 10.0–33.1), respectively.

The T0-only propensity-score analysis yielded ORs of 1.43 (95% CI 0.87–2.35; P = 0.157) in MIMIC-IV and 1.37 (95% CI 0.55–3.42; P = 0.503) in eICU-CRD. The pooled cohort-adjusted OR was 1.42 (95% CI 0.92–2.19; P = 0.118).

# 3 Supplementary tables

**Table S2.** Baseline characteristics in MIMIC-IV

| **Variable** | **No early AVP (n = 791)** | **Early AVP (n = 295)** | **SMD** |
| --- | --- | --- | --- |
| **Demographics** | | | |
| Age, years | 63.00 [54.00, 72.00] | 62.00 [52.00, 74.00] | 0.045 |
| Male sex, n (%) | 474 (59.9) | 186 (63.1) | 0.064 |
| White race or ethnicity, n (%) | 486 (61.4) | 146 (49.5) | 0.242 |
| Weight, kg | 81.00 [69.20, 98.20] | 81.90 [70.00, 96.00] | 0.044 |
| **Liver disease and organ dysfunction** | | | |
| MELD-Na score | 21.63 [14.83, 29.15] | 23.77 [17.13, 29.88] | 0.220 |
| Database-derived organ-dysfunction proxy, n (%) | 647 (81.8) | 283 (95.9) | 0.461 |
| Baseline AKI stage 2–3, n (%) | 86 (17.5) | 33 (19.5) | 0.053 |
| Creatinine, mg/dL | 1.40 [0.90, 2.10] | 1.80 [1.12, 2.60] | 0.168 |
| Total bilirubin, mg/dL | 1.80 [0.70, 4.93] | 2.00 [0.78, 5.93] | 0.024 |
| Albumin, g/dL | 2.90 [2.40, 3.40] | 2.60 [2.10, 3.20] | 0.311 |
| INR | 1.60 [1.30, 2.20] | 1.80 [1.42, 2.30] | 0.116 |
| Platelet count, 10⁹/L | 141.00 [85.00, 216.00] | 133.00 [79.75, 198.25] | 0.067 |
| Sodium, mmol/L | 137.00 [134.00, 141.00] | 138.00 [134.00, 142.00] | 0.112 |
| Lactate, mmol/L | 3.00 [1.80, 5.00] | 5.50 [3.10, 9.10] | 0.647 |
| **Cirrhosis-related complications** | | | |
| Ascites, n (%) | 379 (47.9) | 128 (43.4) | 0.091 |
| Hepatic encephalopathy, n (%) | 118 (14.9) | 35 (11.9) | 0.090 |
| Hepatorenal syndrome, n (%) | 68 (8.6) | 21 (7.1) | 0.055 |
| Spontaneous bacterial peritonitis, n (%) | 45 (5.7) | 24 (8.1) | 0.097 |
| Gastrointestinal or variceal bleeding, n (%) | 226 (28.6) | 75 (25.4) | 0.071 |
| **Shock severity and early ICU treatments** | | | |
| Mean MAP during 0–6 h, mmHg | 72.09 [66.73, 78.11] | 70.88 [65.50, 77.12] | 0.146 |
| Minimum MAP during 0–6 h, mmHg | 60.50 [54.00, 66.00] | 56.00 [50.00, 62.00] | 0.393 |
| Norepinephrine dose at T0, µg/kg/min | 0.10 [0.06, 0.20] | 0.30 [0.20, 0.42] | 0.672 |
| Maximum norepinephrine dose during 0–6 h, µg/kg/min | 0.15 [0.09, 0.28] | 0.42 [0.30, 0.50] | 0.632 |
| Mechanical ventilation, n (%) | 569 (71.9) | 268 (90.8) | 0.501 |
| Albumin use during 0–6 h, n (%) | 160 (20.2) | 74 (25.1) | 0.116 |
| Corticosteroid use during baseline window, n (%) | 71 (9.0) | 60 (20.3) | 0.326 |
| Antibiotic use during baseline window, n (%) | 613 (77.5) | 243 (82.4) | 0.122 |
| **Comorbidities** | | | |
| Chronic kidney disease, n (%) | 123 (15.5) | 46 (15.6) | 0.001 |
| Hypertension, n (%) | 555 (70.2) | 171 (58.0) | 0.256 |
| Diabetes, n (%) | 229 (29.0) | 81 (27.5) | 0.033 |
| Congestive heart failure, n (%) | 230 (29.1) | 74 (25.1) | 0.090 |
| Malignancy, n (%) | 226 (28.6) | 95 (32.2) | 0.079 |

Values are median [interquartile range] or n (%). Percentages use available-case denominators when source data needed to derive a variable were missing. AKI, acute kidney injury; AVP, vasopressin; ICU, intensive care unit; INR, international normalized ratio; MAP, mean arterial pressure; MELD-Na, Model for End-Stage Liver Disease–sodium; SMD, standardized mean difference; T0, first norepinephrine administration.

**Table S3.** Baseline characteristics in eICU-CRD

| **Variable** | **No early AVP (n = 265)** | **Early AVP (n = 91)** | **SMD** |
| --- | --- | --- | --- |
| **Demographics** | | | |
| Age, years | 59.00 [51.00, 67.00] | 57.00 [52.00, 65.00] | 0.104 |
| Male sex, n (%) | 147 (55.5) | 53 (58.2) | 0.056 |
| White race or ethnicity, n (%) | 208 (78.5) | 71 (78.0) | 0.011 |
| Weight, kg | 83.90 [69.05, 102.10] | 86.60 [72.20, 104.00] | 0.112 |
| **Liver disease and organ dysfunction** | | | |
| Database-derived organ-dysfunction proxy, n (%) | 166 (62.6) | 71 (78.0) | 0.342 |
| Baseline AKI stage 2–3, n (%) | 38 (16.0) | 14 (17.1) | 0.030 |
| Creatinine, mg/dL | 1.69 [1.04, 2.94] | 1.92 [1.38, 2.84] | 0.097 |
| Total bilirubin, mg/dL | 3.10 [1.30, 7.30] | 4.40 [2.00, 9.00] | 0.149 |
| Albumin, g/dL | 2.20 [1.80, 2.80] | 2.30 [2.00, 2.80] | 0.020 |
| Platelet count, 10⁹/L | 112.00 [65.75, 189.00] | 107.50 [63.75, 138.75] | 0.301 |
| Sodium, mmol/L | 136.00 [131.00, 141.00] | 137.00 [132.00, 141.00] | 0.097 |
| Lactate, mmol/L | 3.10 [1.80, 6.50] | 7.20 [4.67, 11.03] | 0.623 |
| **Cirrhosis-related complications** | | | |
| Ascites, n (%) | 64 (24.2) | 16 (17.6) | 0.162 |
| Hepatic encephalopathy, n (%) | 55 (20.8) | 17 (18.7) | 0.052 |
| Hepatorenal syndrome, n (%) | 34 (12.8) | 13 (14.3) | 0.043 |
| Spontaneous bacterial peritonitis, n (%) | 0 (0.0) | 0 (0.0) | 0.000 |
| Gastrointestinal or variceal bleeding, n (%) | 26 (9.8) | 11 (12.1) | 0.073 |
| **Shock severity and early ICU treatments** | | | |
| Mechanical ventilation, n (%) | 87 (32.8) | 45 (49.5) | 0.343 |
| **Comorbidities** | | | |
| Chronic kidney disease, n (%) | 148 (55.8) | 59 (64.8) | 0.184 |
| Hypertension, n (%) | 24 (9.1) | 6 (6.6) | 0.092 |
| Diabetes, n (%) | 25 (9.4) | 13 (14.3) | 0.150 |
| Congestive heart failure, n (%) | 18 (6.8) | 9 (9.9) | 0.112 |
| Malignancy, n (%) | 10 (3.8) | 4 (4.4) | 0.031 |

Values are median [interquartile range] or n (%). Percentages use available-case denominators when source data needed to derive a variable were missing. Variables with unreliable availability in eICU-CRD were omitted. AKI, acute kidney injury; AVP, vasopressin; eICU-CRD, eICU Collaborative Research Database; ICU, intensive care unit; SMD, standardized mean difference.

**Table S4.** Primary and secondary outcomes

| **Outcome** | **Dataset** | **No early AVP** | **Early AVP** | **Weighted difference** | **N** |
| --- | --- | --- | --- | --- | --- |
| **Primary outcome** | | | | | |
| New RRT within 7 days | MIMIC-IV | 176/791 (22.3%) | 98/295 (33.2%) | 6.4 pp (−0.6 to 13.9) | 1086 |
| New RRT within 7 days | eICU-CRD | 42/265 (15.8%) | 22/91 (24.2%) | 1.6 pp (−8.7 to 12.6) | 356 |
| New RRT within 7 days | Fixed-effect pooled | 23.8% | 28.9% | 5.1 pp (−1.2 to 10.8) | 1442 |
| **Secondary outcomes** | | | | | |
| 28-day mortality† | MIMIC-IV | 336/791 (42.5%) | 183/295 (62.0%) | 6.3 pp (−1.6 to 13.9) | 1086 |
| 28-day mortality† | eICU-CRD | 121/265 (45.7%) | 62/91 (68.1%) | 13.9 pp (1.0 to 26.8) | 356 |
| ICU mortality | MIMIC-IV | 248/791 (31.4%) | 161/295 (54.6%) | 9.2 pp (1.7 to 17.2) | 1086 |
| ICU mortality | eICU-CRD | 95/265 (35.8%) | 55/91 (60.4%) | 18.6 pp (5.7 to 32.2) | 356 |
| Hospital mortality | MIMIC-IV | 307/791 (38.8%) | 182/295 (61.7%) | 8.2 pp (1.0 to 15.9) | 1086 |
| Hospital mortality | eICU-CRD | 123/265 (46.4%) | 63/91 (69.2%) | 14.6 pp (2.5 to 27.4) | 356 |
| RRT-free days to Day 7‡ | MIMIC-IV | 7.00 (0.18–7.00) | 0.47 (0.00–7.00) | −0.61 (−1.12 to −0.11) | 1086 |
| RRT-free days to Day 7‡ | eICU-CRD | 7.00 (0.00–7.00) | 0.86 (0.00–7.00) | −0.78 (−1.61 to 0.06) | 356 |
| ICU length of stay, days | MIMIC-IV | 4.16 (2.26–8.11) | 3.94 (1.39–10.24) | 0.26 (−0.92 to 1.53) | 1086 |
| ICU length of stay, days | eICU-CRD | 4.00 (2.01–7.86) | 2.36 (1.07–9.54) | −0.09 (−2.12 to 2.17) | 356 |
| Hospital length of stay, days | MIMIC-IV | 11.29 (5.84–20.50) | 9.83 (1.90–20.66) | 1.56 (−0.96 to 4.49) | 1086 |
| Hospital length of stay, days | eICU-CRD | 6.87 (2.86–12.43) | 3.37 (1.12–12.81) | −1.33 (−3.97 to 1.22) | 356 |

Values are unweighted event counts and percentages or median (interquartile range), unless stated otherwise. Weighted difference is early AVP minus no early AVP; binary outcomes are risk differences in percentage points and continuous outcomes are mean differences. †In eICU-CRD, 28-day mortality denotes in-hospital death within 28 days because post-discharge follow-up was unavailable. ‡Patients who died before RRT within 7 days were assigned 0 RRT-free days. AVP, vasopressin; eICU-CRD, eICU Collaborative Research Database; ICU, intensive care unit; pp, percentage points; RRT, renal replacement therapy. For the pooled primary-outcome row, the group columns report pooled overlap-weighted risks.

**Table S5.** Availability of prespecified subgroup variables in MIMIC-IV

| **Variable** | **Available, n** | **Missing, n** | **Missing, %** |
| --- | --- | --- | --- |
| Baseline AKI stage | 661 | 425 | 39.1 |
| MELD-Na score | 669 | 417 | 38.4 |
| Lactate | 818 | 268 | 24.7 |
| Norepinephrine dose at T0 | 1,086 | 0 | 0.0 |
| Mean MAP during 0-6 h | 1,059 | 27 | 2.5 |
| Mechanical ventilation | 1,086 | 0 | 0.0 |
| Database-derived organ-dysfunction proxy | 1,086 | 0 | 0.0 |

Availability counts correspond to the analytic denominators used in the prespecified subgroup analyses. Percentages are calculated using N = 1,086. AKI, acute kidney injury; MAP, mean arterial pressure; MELD-Na, Model for End-Stage Liver Disease-sodium; T0, first norepinephrine administration. Baseline AKI stage and MELD-Na were missing for 39.1% and 38.4% of patients, respectively; subgroup estimates for these variables should be interpreted cautiously.

**Table S6.** Timing of vasopressin initiation among patients with no early vasopressin

| **Dataset** | **No early AVP, N** | **>6–12 h, n (%)** | **>12–24 h, n (%)** | **>24–48 h, n (%)** | **>48 h–Day 7, n (%)** | **No AVP recorded by Day 7, n (%)** | **Later initiation, median (IQR), h** |
| --- | --- | --- | --- | --- | --- | --- | --- |
| MIMIC-IV | 791 | 79 (10.0) | 56 (7.1) | 43 (5.4) | 28 (3.5) | 585 (74.0) | 15.0 (9.7–33.7) |
| eICU-CRD | 265 | 28 (10.6) | 26 (9.8) | 14 (5.3) | 13 (4.9) | 184 (69.4) | 16.2 (10.0–33.1) |

Early-vasopressin patients were, by definition, initiated during 0–6 h (MIMIC-IV, n = 295; eICU-CRD, n = 91). Median (IQR) is calculated among later initiators only. ‘No AVP recorded by Day 7’ denotes no recorded initiation through Day 7 or the end of available follow-up, whichever came first. AVP, vasopressin; eICU-CRD, eICU Collaborative Research Database; IQR, interquartile range.

**Table S7.** Sensitivity analysis using a T0-only propensity-score model

| **Analysis** | **Cohort N** | **Early AVP, n** | **No early AVP, n** | **OR (95% CI)** | **P value** |
| --- | --- | --- | --- | --- | --- |
| MIMIC-IV | 1086 | 295 | 791 | 1.43 (0.87–2.35) | 0.157 |
| eICU-CRD | 356 | 91 | 265 | 1.37 (0.55–3.42) | 0.503 |
| Pooled with cohort adjustment | 1442 | 386 | 1056 | 1.42 (0.92–2.19) | 0.118 |

The propensity-score models used variables available at or before T0 and excluded summaries or treatments measured during the 0–6 h treatment-classification window. Database-specific overlap-weighted estimates are shown together with a pooled outcome model that included cohort as a covariate. Odds ratios greater than 1 indicate higher odds of new RRT with early vasopressin. AVP, vasopressin; CI, confidence interval; OR, odds ratio; RRT, renal replacement therapy; T0, first norepinephrine administration.

# 4 Supplementary figures

**Figure S2.** Covariate balance before and after overlap weighting. Absolute SMDs decreased after weighting in MIMIC-IV. The organ-dysfunction proxy was used for database-specific stratification and does not represent clinically adjudicated ACLF. AVP, vasopressin; MAP, mean arterial pressure; MELD-Na, Model for End-Stage Liver Disease–sodium; NE, norepinephrine; SMD, standardized mean difference.

**Figure S3.** Weighted cumulative incidence of new renal replacement therapy. Curves begin at the 6-h landmark and extend through Day 7 in MIMIC-IV. AVP, vasopressin; RRT, renal replacement therapy.

**Figure S4.** Day-7 outcome states after the 6-h landmark. Bars show weighted proportions alive without RRT, receiving new RRT, or dead before RRT. AVP, vasopressin; eICU-CRD, eICU Collaborative Research Database; RRT, renal replacement therapy.

**Figure S5.** Subgroup analyses in MIMIC-IV. Odds ratios compare early vasopressin with no early vasopressin. The organ-dysfunction proxy was a database-derived stratification variable rather than a clinically adjudicated ACLF diagnosis. AKI, acute kidney injury; AVP, vasopressin; CI, confidence interval; MAP, mean arterial pressure; MELD-Na, Model for End-Stage Liver Disease–sodium; NE, norepinephrine; RRT, renal replacement therapy.

**Figure S6.** Sensitivity analyses for new renal replacement therapy. No analysis produced a statistically significant renal-benefit estimate. The row labelled ‘Alternative adjusted model’ uses the alternative sensitivity specification and is distinct from the conventional multivariable model in Table 1. The organ-dysfunction-proxy restriction does not represent a clinically adjudicated ACLF cohort. AVP, vasopressin; CI, confidence interval; CKD, chronic kidney disease; GI, gastrointestinal; IPTW, inverse probability of treatment weighting; RRT, renal replacement therapy.

**Figure S7.** Timing of first vasopressin initiation after T0. All early-vasopressin patients initiated during 0–6 h by definition. Among patients with no vasopressin during 0–6 h, the stacked bars show later initiation windows and the proportion with no vasopressin recorded by Day 7. AVP, vasopressin; eICU-CRD, eICU Collaborative Research Database; T0, first norepinephrine administration.

**Figure S8.** T0-only propensity-score sensitivity analysis. Propensity-score models used variables available at or before norepinephrine initiation. Database-specific overlap-weighted ORs and the pooled cohort-adjusted estimate are shown. Values greater than 1 indicate higher odds of new RRT with early vasopressin; the dashed line indicates OR = 1. CI, confidence interval; eICU-CRD, eICU Collaborative Research Database; OR, odds ratio; RRT, renal replacement therapy; T0, first norepinephrine administration.
